# Dysregulation of metabolites and high-altitude illnesses development under plateau conditions

**DOI:** 10.3389/fphys.2025.1600374

**Published:** 2025-07-02

**Authors:** Xue Bai, Yujuan Qi, Qiang Zhang

**Affiliations:** ^1^ College of Pharmacy, Qinghai Minzu University, Xining, Qinghai, China; ^2^ Qinghai Provincial People’s Hospital, Xining, Qinghai, China

**Keywords:** high-altitude illness, metabolite, pathogenesis, hypoxia, plateau environment

## Abstract

**Background:**

Under plateau conditions, changes in metabolite levels can affect cellular signal transduction, protein activity, and gene expression, thereby inducing a series of physiopathological changes. Altered metabolite signaling in plateau environments may be associated with the onset and progression of high-altitude illnesses. This review summarizes the effects of a plateau environment on diseases, the mechanism of occurrence of such high-altitude illnesses, and the regulation of diseases by metabolites and proposes the role of metabolites in regulating high-altitude illnesses in plateau environments.

**Main body:**

Through a systematic review and analysis of the existing literature, we propose and focus on an original theoretical framework for the metabolic regulation of high-altitude illnesses. We hypothesize that the development of high-altitude illnesses is not merely a reflection of passive changes in metabolites but rather stems from an imbalance in a recognizable and intervenable metabolic regulatory network.

**Conclusion:**

Metabolites have very important roles and implications in high-altitude illnesses, and a better comprehension of the regulatory role of metabolites in the pathogenesis of high-altitude illnesses can provide theoretical support for the prevention and treatment of high-altitude illnesses.

## 1 Introduction

Plateau terrain is widely distributed globally, accounting for approximately 45% of the total land area. This terrain is characterized by low pressure and low oxygen, strong radiation, strong sunshine, and low temperature, of which low oxygen is the main factor affecting human life activities (Xu et al., 2023). At an altitude ≥2,500 m, the body’s metabolic processes will undergo a series of changes due to the lower atmospheric pressure, leading to tissue hypoxia. Glycolysis, the tricarboxylic acid cycle (TCA), amino acid metabolism, and other pathways affect the expression of proteins and genes involved in the transport of oxygen, blood vessel growth, erythropoiesis, and energy metabolism (Hartman-Ksycinska et al., 2016). The body may experience acute mountain sickness (AMS), high altitude pulmonary edema (HAPE), high altitude cerebral edema (HACE), and chronic mountain sickness (CMS). Furthermore, other factors in the plateau environment, such as severe cold, strong winds, and intense radiation, can increase the incidence of high-altitude illnesses.

Metabolites exist both inside and outside cells and maintain their activities. Metabolites act on proteins by non-covalent binding or covalent post-translational modifications to regulate various biological processes inside and outside the cell (Russell and Camargo, 2022). An increasing number of studies are reporting that diabetes, cancer, cardiovascular diseases, etc., are all invariably associated with metabolite dysregulation (Li et al., 2022). Under the high-altitude hypoxic environment, cells undergo metabolic reprogramming to adapt to the special environment, while metabolites undergo significant changes, which may increase the risk of developing high-altitude illnesses (Zhou et al., 2024). However, the mechanisms of metabolite-mediated high-altitude illnesses under plateau conditions remain unelucidated because they are multi-factorial and relatively complex. This review describes the occurrence and pathogenesis of high-altitude illnesses in plateau environments and the effects of metabolites on diseases, highlights speculations on the relationship between metabolite alterations and common high-altitude illnesses, and summarizes the mechanisms of occurrence of common high-altitude illnesses caused by metabolite alterations to provide a theoretical basis for their prevention and treatment.

## 2 Diseases caused by plateau environments

The plateau has a unique geographical environment characterized by low air pressure, low oxygen levels, intense radiation, and low temperatures, with low oxygen being the most critical element affecting human life activities (Witt and Huerta-Sanchez, 2019). A series of physiological mechanisms are triggered after hypoxia, i.e., the acclimatization and adaptation to the hypoxia environment, to improve the transportation of O_2_ and the utilization of O_2_ by the organism, especially the regulation of the respiratory system, the blood system, and the cardiovascular system (Leissner and Mahmood, 2009). The typical physiological effects of plateauing include acute hypoxic ventilatory response, hypoxemia, excessive erythrocytes, interstitial and/or alveolar edema, and edema of brain tissue or brain cells (Basnyat, 2000). AMS, HAPE, and HACE may develop in plains populations moving to the plateau, whereas diseases affecting permanent residents of the plateau or the indigenous highland populations include CMS and high-altitude pulmonary hypertension (Gatterer et al., 2024).

When an organism rapidly enters a plateau, hypoxia stimulates peripheral chemoreceptors, which in turn excite the respiratory center and trigger the hypoxic ventilatory response (Basu et al., 1996). This response causes breathing to become deeper and faster, improving pulmonary ventilation. It can be sustained for approximately 8 weeks in persistent hypoxia (Pamenter and Powell, 2016). With prolonged exposure to the plateau environment, an organism can efficiently adapt and promote oxygen transport and utilization by increasing respiratory membrane area, shortening gas exchange distance, and appropriately elevating hemoglobin (Hb) levels. However, hyperventilation can lead to respiratory alkalosis, the recovery time of which is prolonged with altitude (Bartsch and Saltin, 2008). This respiratory compensation and adjustment is directly linked to oxygen uptake, which in turn profoundly affects the adaptation of the blood system. Previous studies have shown that high-altitude hypoxia promotes erythropoietin (EPO) synthesis in the kidney and liver as well as alterations in the bone marrow microenvironment, which promotes the maturation and proliferation of erythroid progenitor cells, thereby orchestrating a cell-type-specific hypoxic response (Haase, 2013). Simultaneously, hypoxia increases 2,3-diphosphoglycerate production, decreases the affinity of Hb for oxygen, and shifts the oxygen dissociation curve to the right, facilitating oxygen release from tissues, which is an important adaptive response (Savourey et al., 2004). However, prolonged exposure to high-altitude hypoxia can lead to excessive erythrocyte proliferation and increase blood viscosity, triggering CMS, the prevalence of which increases with altitude (Duhm and Gerlach, 1971). Changes in the oxygen-carrying and oxygen-releasing capacity of the blood will inevitably require the cardiovascular system to adjust accordingly. When first arriving at a plateau, the body’s heart rate and cardiac output increase significantly, and a slight rise in blood pressure, mainly from arterial chemoreceptor excitation and increased sympathetic tone (Shirai et al., 1985). After several days of acclimatization, cardiac output recovers, but the heart rate is still fast, the output per beat decreases, and ventricular function is maintained (Naeije, 2010). Simultaneously, hypoxic pulmonary vasoconstriction as a physiologic compensatory response leads to a dramatic increase in pulmonary arterial pressure. Pulmonary hypertension is prevalent in migrants, and chronicity can lead to smooth muscle hyperplasia of small pulmonary arteries, wall thickening and structural reconstruction of the pulmonary vasculature, and, in severe cases, progression to HAPE and right heart dysfunction (Sydykov et al., 2021).

The neurological effects of high-altitude hypoxia are particularly pronounced and can lead to confusion, fatigue, and sleep disturbances. Extreme hypoxic exposure can lead to impaired reasoning and judgment, an effect that can last for several days and may not be recovered even after returning to lower altitudes (Leissner and Mahmood, 2009). Given that the brain consumes approximately 20% of the body’s oxygen at rest, the brain is highly sensitive to hypoxemia and optimizes oxygen transport by regulating cerebral circulation. At a high-altitude hypoxic environment, hypoxia-induced cerebral vasodilatation is partially counteracted by hypobaric pressure-induced cerebral vasoconstriction, which ultimately results in only a modest increase in cerebral blood flow, and the increase is proportional to the degree of hypoxemia (Hartman-Ksycinska et al., 2016). Besides the above systems, the plateau environment also has an impact on the digestive system, leading to intestinal mucosal damage, atrophy, and barrier dysfunction, accompanied by changes in gut microbiota (Xing et al., 2018; Bai et al., 2022). In maintaining the homeostasis of the internal environment, the kidneys play a key role in plateau habituation and the prevention of high-altitude illnesses by regulating body fluids, electrolytes, and acid-base balance (Goldfarb-Rumyantzev and Alper, 2014).

It is precisely because the low-oxygen environment of the plateau has numerous effects on various systems, tissues, and organs of the human body, which may lead to different degrees of high-altitude illnesses. Studies have shown that the body’s oxygen saturation usually remains above 90% as compensatory ventilation increases with decreasing exercise capacity at moderate altitudes (1,500–2,500 m). For most susceptible individuals, AMS generally occurs above 2,500 m above sea level, whereas most high-altitude illnesses occur in a plateau environment at high altitude (2,500–4,500 m), where oxygen saturation may be <90% and worsening of hypoxemia occurs during exercise and sleep. When the body is at a very high altitude (4,500–5,500 m), the incidence of HAPE and HACE increases, while the body requires a period of acclimatization to prevent the onset of plateau disease. When in extremely high altitudes, i.e., altitude >5,500 m, the body experiences significant hypoxemia and hypocapnia, and hypoxic stress leads to progressive physiological deterioration and ultimately breaks the body’s adaptive capacity (Davis and Hackett, 2017). Among individuals who have lived at altitudes >2,500 m for significant periods of time, the body loses its habituation to a plateau environment, thus causing CMS, which manifests itself as erythrocytosis and severe hypoxemia, often accompanied by moderate or severe pulmonary hypertension, which may eventually evolve into pulmonary heart disease and cause congestive heart failure (Villafuerte and Corante, 2016). CMS onset is closely related to altitude, gender, race, and other factors. The clinical manifestations disappear when moving to lower altitudes and recur when returning to higher altitudes. Furthermore, living at high altitudes has some benefits, including reduced rates of obesity, diabetes, and coronary heart disease, which may be attributed to the effects of altitude and dietary differences in highland areas (Hurtado et al., 2012).

## 3 Regulatory mechanisms of high-altitude illnesses

The regulatory mechanism of high-altitude illnesses is a complex pathological process induced by hypoxia that spans physiological, molecular, and cellular levels and is centrally characterized by the activation of oxidative stress mechanisms, the regulatory roles of key transcription factors, and dynamic changes in epigenetic modifications.

### 3.1 Oxidative stress

The core adaptive mechanism triggered by the plateau environment starts with the cells’ ability to sense oxygen. Almost all cells sense a decrease in oxygen partial pressure and initiate an adaptive response via specific receptors and signaling pathways. However, in the unique environment of the plateau, this dysregulation of the oxygen-sensing response plays a key role in the development of high-altitude illnesses. Notably, the plateau environment itself is a multiple stressors (low pressure, hypoxia, and intense radiation), which interferes with the normal functioning of the oxygen-sensing system and collectively leads to the overproduction of free radicals (e.g., hydrogen peroxide and superoxide anion), which exceeds the antioxidant system’s scavenging capacity and thus triggers significant oxidative stress. Oxidative stress leads to key pathological changes, such as extensive lipid peroxidation, protein denaturation, and DNA damage (Brand, 2016). This is an important pathogenic mechanism for high-altitude illnesses. Studies of patients with AMS have found that free radical-mediated neurogenic damage is the main cause of altered blood-brain barrier permeability and inflammatory responses (Bailey, 2004). In HAPE, it manifests as oxygen-free radical damage to the pulmonary vascular endothelium, which increases permeability and leads to hyperosmolar pulmonary edema (Bailey et al., 2001). In CMS, it is associated with persistent free radical accumulation and tissue damage due to tissue hypoxia (Jefferson et al., 2004). Therefore, abnormal oxygen sensing and oxidative stress injury are interconnected and collectively constitute the core pathophysiological network underlying the development of high-altitude illnesses.

### 3.2 Transcription factor

Hypoxia-inducible factor (HIF) serves as a core transcriptional regulator of the hypoxia response, and its subunits HIF-1α and HIF-2α play key roles in the development of high-altitude illnesses (Kaelin and Ratcliffe, 2008; Cummins et al., 2020). Acute hypoxia activates HIF, which mediates the cellular adaptive response to hypoxia by up-regulating the transcription of its target genes, such as EPO and glycolytic enzymes (du Souic et al., 2011). However, persistent activation of hypoxia-induced gene programs can lead to pathological changes (Lee et al., 2020). Sustained hypoxia stabilizes HIF-1α and HIF-2α expression to positively regulate vascular endothelial growth factor (*VEGF*) expression. Elevated VEGF levels are positively correlated with zonula occludens-1, junctional adhesion molecules, claudin 4, and claudin 5 expression, suggesting that hyperactivation of HIF-1α and HIF-2α can trigger increased intracranial pressure, increased vascular permeability, and disruption of the blood-brain barrier, ultimately leading to HACE (Turner et al., 1985; Sarada et al., 2015). In HAPE, HIF-1α plays a central role in regulating lung vascular remodeling-related genes such as BCL2 interacting protein 3-like, angiopoietin-like 4, and egl-9 family hypoxia-inducible factor 1 (*EGLN1*) (Yuhong et al., 2018; Soree et al., 2016). Its overexpression also directly activates the isthmin 1 gene, which may be an important regulator of hypoxia-induced endothelial hyperpermeability (Li et al., 2021). Furthermore, HIF-1α influences CMS development by modulating NADPH oxidase 4 and peroxisome proliferator-activated receptor-γ expression (Li S. et al., 2024). HIF-2α is pivotal in HIF-EPO-induced erythrocyte hyperplasia and VEGF-VEGFR-mediated angiogenesis, thereby impacting CMS (Li K. et al., 2024).

Besides HIF, other hypoxia-sensitive transcription factors play an equally important role. For instance, nuclear factor erythroid 2-related factor 2 (Nrf2) influences the pathological process of various high-altitude illnesses (Cadenas, 2018). Nuclear factor-κB (NF-κB) activation is closely associated with endothelial inflammatory injury, increased pulmonary capillary permeability, and the formation of HACE and HAPE (Geng et al., 2022; Shi et al., 2021). p53 may influence the course of AMS and HAPE by regulating cell survival and apoptosis (Azad et al., 2022; Sun et al., 2024). Activator protein-1 (AP-1) is involved in CMS by regulating cell proliferation, differentiation, apoptosis, angiogenesis, and metabolism (Xi et al., 2004; Nanduri and Nanduri, 2007). Sentrin-specific protease 1, serum- and glucocorticoid-regulated kinase 3, COP9 signalosome subunit 5, PR domain zinc finger protein 1, and intraflagellar transport 122 are also involved in CMS pathogenesis (Stobdan et al., 2017).

The regulation of transcription factors in high-altitude illnesses is not isolated but is the result of the synergistic action of multiple factors. The interaction of HIF with factors such as Nrf2, NF-κB, p53, AP-1, and others is crucial in hypoxic stress, inflammatory response, and damage repair. For example, hypoxia activates HIF-1α and Nrf2, promoting their nuclear translocation, and these transcription factors synergistically regulate the expression of oxidative stress-related genes (Bae et al., 2024; Malacrida et al., 2019). There are signaling pathway interactions between HIF-1α and AP-1, such as the phosphatidylinositol 3-kinase and mitogen-activated protein kinase pathways, which synergistically promote angiogenesis to improve oxygenation but may also trigger excessive inflammatory responses, affecting the clinical performance of high-altitude illnesses (Wei and Zhang, 2024; Kunz and Ibrahim, 2003; Kim et al., 2006). NF-κB and AP-1 can also interact with each other to regulate their own activities, and together, they can exacerbate the inflammatory response in the lungs or brain, aggravating the symptoms of HAPE or HACE (Jutz et al., 2016; Yun and Lee, 2023).

### 3.3 Epigenetic factors

Epigenetic factors, including DNA methylation, non-coding RNA, and histone modification, are closely related to the development of high-altitude illness. Hypermethylation of the *EGLN1* gene in patients with CMS was found to be associated with excessive erythropoiesis, while hypomethylation of the gene may increase the risk of HAPE by decreasing plasma Prolyl hydroxylase domain 2 levels and oxygen saturation (Julian, 1985; Sharma et al., 2022). Studies in patients with HAPE further revealed significant aberrant methylation patterns in the CpG island of the *APELLIN* gene and in the promoter regions of the *CYP2S1* and *CYP39A1* genes, suggesting that they may serve as potential molecular markers for assessing susceptibility to HAPE and predicting disease progression (Wang et al., 2024; Mishra et al., 2015; Jin et al., 2022; Wang et al., 2023). Furthermore, acute hypoxia exposure decreased LINE-1 methylation levels and elevated EPAS1 (encoding HIF-2α) methylation levels, whereas chronic hypoxia showed the opposite trend, highlighting the high sensitivity of epigenetic regulation to the duration of hypoxia exposure and its complexity (Childebayeva et al., 2019; Childebayeva et al., 2021).

Non-coding RNAs play an important role in key pathological mechanisms, including oxidative stress, vascular remodeling, and erythropoiesis, acting as epigenetic regulators. MicroRNAs (miRNAs), which are typical endogenous non-coding RNAs, target and regulate gene networks associated with hypoxic adaptation (Lenkala et al., 2014). A study of AMS found that the expression of several miRNAs, including miR-369-3p, miR-449b-3p, miR-136-3p, and miR-4791, differed (Liu et al., 2017). These miRNAs target genes that are closely related to processes such as ammonia transport and nitrogenous material production. This affects cellular stress responses and metabolic regulation (Beall et al., 2012). A study using a hypoxia animal model also found 57 differentially expressed miRNAs in rat lung tissues, 19 of which were related to the VEGF/Notch signaling pathway. This suggests that miRNAs are involved in regulating hypoxia-induced HAPE (Cai et al., 2020). Additionally, long non-coding RNAs (lncRNAs) and circular RNAs (circRNAs) play a similarly significant role in regulating the expression network of susceptibility genes associated with high-altitude illnesses. In patients with CMS, the lncRNA HIKER/LINC02228 was found to promote erythropoiesis and exacerbate the pathological process of CMS via the casein kinase CSNK2B (Jha et al., 2021). Studies on circRNAs have revealed that circRNAs are enriched under low oxygen conditions at high altitudes and are mainly associated with metabolic pathways such as glycosaminoglycan degradation and pentose-glucuronic acid crossover. They have the potential to be used as disease-prognostic markers (Ge et al., 2021). Histone modifications regulate gene expression by altering chromatin structure and affecting transcription factor binding. Key histone marks (e.g., H4K16Ac, H3K4me3, H3K27ac, H3K9me3, H3K9ac in the promoter region) regulate the expression of the NOX family of genes (*NOX1*, *NOX2*, *NOX4*, *NOX5*), which affects the pathological process of HAPE (Chen et al., 2016; Manea et al., 2018). Histone acetyltransferase p300 relaxes chromatin by acetylating histones; it enhances the binding capacity of transcription factors, which in turn upregulates the expression of HIF-1α and ultimately affects the physiopathological processes associated with plateau diseases, such as angiogenesis, erythropoiesis, and metabolic adaptation (Shi et al., 2023).

In summary, oxidative stress, transcriptional regulation, and epigenetic modifications play a central role in the development of high-altitude illnesses. Notably, these complex regulatory processes do not exist in isolation; they are profoundly influenced by the intracellular and extracellular environments. Among them, the cellular metabolic state and the specific metabolites it produces are key upstream signals that regulate the activity of transcription factors, as well as the availability of substrates for epigenetic modifications. Therefore, a deeper understanding of how metabolites integrate environmental signals and precisely regulate these core mechanisms is essential to unraveling the complete pathophysiological picture of high-altitude illnesses.

## 4 Mechanisms of action of metabolites in regulating disease

Metabolites, the intermediate or end products of cellular metabolism, are present both inside and outside the cell and play important roles in the physiological processes of cells, tissues, and entire organisms. Metabolites can be broadly classified into basal metabolites, intermediate metabolites, and toxic metabolites. Basal metabolites are essential molecules generated from the conversion of nutrients and are mainly classified as sugar metabolites (glucose, fructose), lipid metabolites (fatty acids, ketone bodies, ceramides), and amino acid metabolites (taurine, phenylalanine, tryptophan) (Hoyas and Leon-Sanz, 2019). Intermediate metabolites are intermediate products in cellular metabolic pathways that play key roles in the metabolic process, including TCA cycle intermediates such as succinate, α-ketoglutarate, lactate, etc (Martinez-Reyes and Chandel, 2020). Toxic metabolites are by-products of metabolic processes that usually have toxic effects on the body. Advancements in analytical techniques have revealed the functions of an increasing number of metabolites. Particular attention has been paid to lactic acid, 2-hydroxyglutaric acid, α-ketoglutaric acid, short-chain fatty acids, and tryptophan, which are not only potential disease markers but have also been indicated as causative factors in various pathological conditions (Wen and Duffy, 2017).

### 4.1 Covalent modification of metabolites

Metabolites drive the key covalent chemical modifications of DNA, RNA, and proteins. The dynamic nature of these chemical modifications significantly impacts cellular function and disease occurrence (Rinschen et al., 2019). S-adenosylmethionine (SAM) is an important intermediate metabolite that serves as a core methyl donor in intracellular methylation reactions. SAM plays a pivotal role in DNA methylation, impacting chromatin status, gene expression, and genome stability throughout embryogenesis and the life cycle (Kundaje et al., 2015; Petkovich et al., 2017). Metabolites such as threonine, glycine, SAM, and pyruvate can act as cofactors for RNA modification, functioning as sensors and converters of metabolic signals to regulate metabolic rates, tRNA function, and protein synthesis (Helm and Alfonzo, 2014). Post-translational modifications of proteins are also influenced by their metabolites. For example, palmitic acid, itaconic acid, lactic acid, butyric acid, acetone, and other metabolites all serve as donors for the posttranslational modification, including palmitoylation, alkylation, lactylation, and ubiquitylation (Zhu et al., 2022). Palmitic acid, an important intermediate in fatty acid metabolism, participates in the lipid modification of proteins by covalently binding to cysteines near protein membranes. This process is important in the progression of cancer and neuropsychiatric diseases (Rana et al., 2018). Itaconic acid directly alkylates cysteine residues on kelch-like ECH-associated protein 1, negatively regulating NRF2 expression. This regulation changes antioxidant and anti-inflammatory gene expression and exacerbates inflammatory diseases (Mills et al., 2018). As the end product of glycolysis, lactic acid influences gene transcription by altering the spatial conformation of histone proteins, thereby affecting the expression levels of key proteins and genes in diseases such as intestinal disorders, brain disorders, and tumors (Li H. et al., 2024; Zhang et al., 2019). Additionally, metabolites such as short-chain fatty acids and ketone bodies affect protein homeostasis through ubiquitination. These processes are involved in immune responses, inflammatory responses, oxidative stress, fibrosis, and protein degradation in cells (Capone et al., 2023).

### 4.2 Non-covalent binding of metabolites

Besides covalent modifications, non-covalent binding is an important mechanism by which metabolites regulate gene and protein expression. As endogenous ligands, metabolites influence intracellular gene expression, protein function, and cellular signaling pathways by binding to specific receptors, such as G protein-coupled receptors (GPCRs) and nuclear receptors (NRs).

GPCRs, the largest superfamily of receptors on the cell surface, are involved in the regulation of various physiological processes, including neurotransmission, immune responses, and vasodilation (Chen H. et al., 2023). On the one hand, metabolites are sensed by GPCRs, and the corresponding GPCRs are activated, thus playing a role similar to that of neurotransmitters and hormones (Engelstoft et al., 2008). Within the gastrointestinal tract, GPCRs can act as enteral nutrient uptake sensors, mediating responses to ingested carbohydrates, proteins, lipids, and non-nutrients (Psichas et al., 2015). On the other hand, certain metabolites selectively bind to GPCRs, regulating the host immune response and metabolic activities. For example, lactic acid, a small-molecule carboxylic acid metabolite produced by intermediary metabolism, can bind to GPR81, activating it to promote angiogenesis, immune evasion, and chemoresistance and contributing to tumor-induced malignancy (Liu et al., 2024). Similarly, lithocholic acid, a secondary bile acid metabolized by the gut microbiota, activates the GPBAR1-mediated signaling pathway, inhibiting protein degradation and promoting protein synthesis, myogenesis, and skeletal muscle regeneration (Sun et al., 2023). Notably, certain metabolites do not have only one target GPCR, and the same metabolite can be recognized by different GPCRs. Butyric acid, one of the most abundant short-chain fatty acids in the intestine, activates GPCRs such as GPR41, GPR43, and GPR109A (Koh et al., 2016; Husted et al., 2017). Through GPR41 activation, butyric acid modulates sympathetic excitation, regulating energy expenditure and maintaining metabolic homeostasis (Kimura et al., 2020). GPR43, primarily expressed in enterocytes, butyric acid stimulates the release of peptide YY from L cells through the activation of GPR43, which in turn inhibits gastrointestinal motility and increases the release of glucagon-like peptide-1 from enteroendocrine L cells, thereby regulating intestinal function and glucose metabolism and mediating diseases such as diabetes mellitus (Brown et al., 2003). Butyric acid is also a candidate ligand for GPR109A, which activates the inflammatory pathway in colonic macrophages and dendritic cells, induces the differentiation of regulatory T cells and interleukin 10-producing T cells, and increases the secretion of interleukin 18 (Chen et al., 2018).

Similar to GPCRs, metabolites can also act as ligands for NRs, such as peroxisome proliferation-activated receptors and farnesoid X receptors. By directly activating signaling pathways, including AMPK, PI3K/Akt, and MAPK, metabolites regulate hepatic gluconeogenesis, glycogen synthesis, insulin sensitivity, and energy metabolism homeostasis *in vivo*. These pathways are implicated in mediating various diseases such as hepatic injury, obesity, type 2 diabetes mellitus, and hyperlipidemia (Copple and Li, 2016; Weikum et al., 2018).

Metabolites can further bind to the active or inactive sites of enzymes, thereby inhibiting or activating their activities. Histone deacetylases (HDACs) are a family of proteases, including types Ⅰ, Ⅱa, Ⅲb, and Ⅳ, that play essential roles in chromatin structure modification and gene expression regulation (Cheng et al., 2024). Butyric acid was the first HDAC inhibitor identified and was shown to exert anti-inflammatory effects by inhibiting HDAC, reducing lipopolysaccharide-induced macrophage secretion of inflammatory factors, and decreasing intestinal macrophage reactivity (Cousens et al., 1979). Butyric acid deficiency is associated with altered gene transcription and immune regulatory dysfunction, potentially leading to various inflammatory and immune system disorders (Liu et al., 2018). In addition to butyric acid, propionic acid reduces the activation of HDAC6 and HDAC9 in regulatory T cells in mice (Vinolo et al., 2011). Moreover, β-hydroxybutyric acid also functions as an endogenous HDAC inhibitor, playing a key role in regulating metabolism and aging-related diseases (Newman and Verdin, 2014).

**FIGURE 1:**
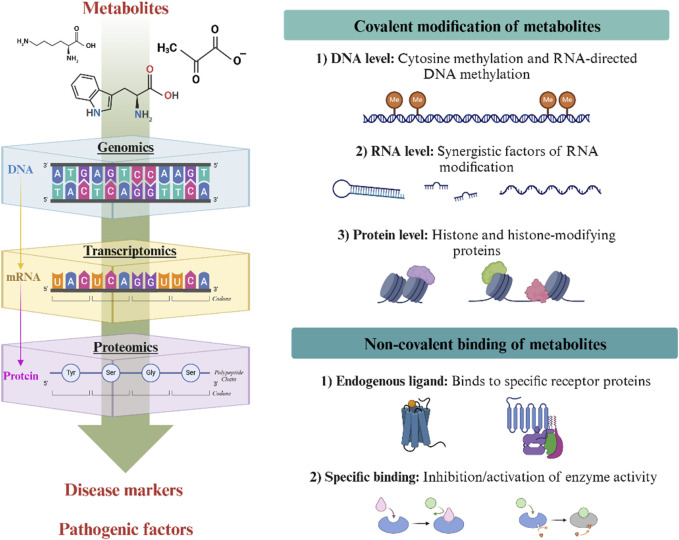
Mechanisms underlying the regulation of metabolites in disease. Metabolites affect cells in two ways: through covalent modifications (key chemical modifications that influence DNA, RNA and proteins) and non-covalent binding (where they act as endogenous ligands). These processes regulate gene expression, protein function and cell signalling pathways.

In summary, metabolites affect cells through both covalent modifications and non-covalent binding to regulate gene expression, protein function, and cellular signaling pathways (Figure 1). The abundance of metabolite-regulated genes and proteins is directly influenced by the metabolic state of the cell and is highly sensitive to external environmental factors. Therefore, an in-depth exploration of the role of metabolites under specific physiological and pathological conditions is important for a better understanding of the relationship between metabolism and disease, as well as for the discovery of new therapeutic strategies and biomarkers.

## 5 Altered metabolite and the development of high-altitude illness

### 5.1 Trends in metabolite dynamics in the plateau environment

There is a significant association between environmental factors and metabolite changes, especially in the special environment of the plateau. Metabolome sequencing of Black Tibetan and Chaka sheep, endemic to the Tibetan Plateau, revealed that the main metabolites included thirty organic acids, nine amino acids, five peptides, four amides, three adenosines, two amines, and other compounds. These findings suggest that the increased concentrations of organic acids and amino acids are related to the survival of these sheep in the plateau’s low-temperature and low-oxygen environment (Guo et al., 2022). Plateau pikas inhabit plateau grasslands and alpine desert steppe zones at altitudes of 3,100–5,100 m. Sequencing of the serum metabolites of plateau pikas at different altitudes revealed differences in the levels of dihydrotestosterone, homoarginine, α-ketoglutarate, 5-hydroxytryptophan and threonine, which may be related to their adaptation to the high-altitude environment (Chen X. et al., 2023). Huang et al. (2022) identified 63 differentially expressed metabolites among the serum metabolites of yaks compared with low-altitude dairy cows. Functional analysis showed that the differentially expressed metabolites were associated with innate immune activation, oxidative stress-related metabolism, and energy metabolism in yaks, indicating that these metabolites play an important role in high-altitude acclimatization.

Compared with changes in the levels of animal metabolites, metabolite alterations induced by the plateau environment in human populations exhibit more complex, multilevel regulatory features. Their metabolic profiles directly reflect environmental adaptive evolutionary pressures. The plasma metabolite distribution characteristics of healthy plateau populations showed that the concentrations of linoleic acid, 12,13-DiHOME, and free fatty acids were significantly higher than those of plains populations, while multiple lysophosphatidylcholines were significantly reduced, suggesting that the lipid energy storage pattern was transformed towards efficient energy supply (Liao et al., 2016). This phenomenon was further enhanced in high-altitude mountaineers, whose synergistic elevation of plasma glutamine and fatty acids enhanced hypoxic tolerance by activating antioxidant pathways and reprogramming energy metabolism (Yin et al., 2025). Furthermore, population-specific metabolic adaptation strategies were evidenced at the level of gene-metabolism interactions. EPAS1, EGLN1 and PPARA gene variants in the Tibetan population inhibit fatty acid oxidative activity, while the levels of branched-chain amino acids, such as valine, leucine and isoleucine, are reduced, suggesting a preferential oxidative energy supply via branched-chain amino acids to optimize oxygen utilization (Ge et al., 2012). The spatiotemporal dynamics of metabolic reprogramming are particularly significant in cross-population comparisons. Plateau-migrating Han Chinese showed metabolic features of enhanced glycolysis, TCA cycling, and elevated NADPH oxidase activity compared to Tibetans, who effectively suppressed oxidative stress through high *HMOX1* gene expression (Zheng et al., 2023). European-descended migrants from the Andean highlands, conversely, have evolved an endothelial barrier protection mechanism of elevated sphingosine phosphate (S1P) levels-S1PR1 receptor activation (Weinstein et al., 2024). Notably, metabolic adaptation is also reversible. Significant perturbations in polyunsaturated fatty acid metabolism in plateau-acclimatized patients returning to the plains reveal a dynamic response of the metabolome to hypoxic acclimatization disorders (Tan et al., 2024).

Moreover, most studies on metabolites influenced by plateau environments have focused on gut microbiota metabolites. For example, the content of bound bile acids increased significantly after acute hypoxia, while free bile acid content decreased, suggesting that the conversion of bound bile acids to free bile acids was impeded following acute exposure to high altitudes (Canniff et al., 1985). Additionally, prolonged exposure to high altitudes led to increased short-chain fatty acid production, which may contribute to host acclimatization to these conditions (Han et al., 2024). Zhao et al. (2024) further showed that butyrate may be an important functional metabolite during high-altitude acclimatization. Exposure to plateau hypoxia remodels intestinal uric acid metabolism. Short-term exposure leads to significantly lower uric acid levels, while long-term residents maintain low levels that negatively correlate with the abundance of key uric acid-degrading bacteria, such as *Lachnospiraceae* (Su et al., 2024). Thus, the plateau environment significantly impacts the production and function of key metabolites, such as bile acids, short-chain fatty acid, and uric acid, by altering the composition and metabolic pathways of the gut microbiota.

### 5.2 High-altitude illness related specific metabolites

Changes in metabolites in patients with high-altitude illness are more strongly characterised by disturbances and are potentially more specific to the disease than those observed in plateau exposure alone. Specifically, plasma metabolites such as hypoxanthine, cysteine and D-arabinitol are significantly elevated in patients with AMS, suggesting disorders of purine metabolism and increased oxidative stress (Zhu et al., 2015). These differential metabolites can serve as potential AMS biomarkers. Conversely, HAPE exhibits characteristic amino acid metabolism disorders, with increased plasma concentrations of branched-chain amino acids (e.g., valine, leucine, and glutamine) by 40%–60%. In HAPE, the concentrations of branched-chain amino acids (e.g., phosphatidylcholine and sphingomyelin) decreased markedly, reflecting the compensatory metabolic state of accelerated muscle proteolysis and inhibited liver gluconeogenesis (Luo et al., 2012). Notably, study further found that a combined assay of C8-ceramide, sphingomyelin, and glutamine has a high diagnostic value for HAPE (Li et al., 2015). Blood exosomal metabolomics studies of HACE revealed 26 significantly altered metabolites, including (+)-patchouli acid, choline, adenosine, adenosine 5′-phosphate, deoxyguanosine 5′-phosphate, guanosine, and hypoxanthine-9-β-D-arabinofuranoside. Abnormal elevation of hypoxanthine derivatives, choline, and adenosine metabolites is closely associated with blood-brain barrier disruption and energy crisis, and these metabolites can likewise be used as potential biomarkers of HACE (Tang et al., 2024).

Compared with acute high-altitude illness, the metabolic profile of CMS more accurately reflects the body’s adaptive or pathological changes to chronic hypoxia and is more complex. The accumulation of specific metabolites, such as fumaric acid, inosine, phytanic acid, and ribose, in the serum of patients with CMS, alongside decreased levels of sulphate, oxalate, and glutamine, collectively suggests a combined effect of mitochondrial dysfunction, antioxidant defence exhaustion, and urea cycle disruption (Chang et al., 2019). Notably, the compensatory downregulation of the pentose phosphate pathway is closely associated with the development of CMS. Inhibiting the pentose phosphate pathway reduces the production of the key coenzyme NADPH, impairing the regenerative capacity of reduced glutathione and thereby exacerbating oxidative stress injury (Wang et al., 2019). However, upregulation of the pathway through the supplementation of intermediates such as ribitol effectively alleviates symptoms, fully confirming the centrality of the pentose phosphate pathway in maintaining energy metabolic homeostasis and redox balance. Additionally, *Lachnospiraceae* enriched in the intestinal tract of plateau populations produce butyrate, which can help alleviate hypoxia-induced intestinal barrier damage and cellular lactate accumulation, as well as blunting the excessive erythrocytosis response. Long-term chronic hypoxia, in turn, weakens aerobic metabolism, leading to a decrease in the gut microbiota metabolite response and resulting in a vicious cycle of “hypoxia–microbiota disruption–metabolic abnormality” (Su et al., 2024).

Patients with high-altitude illness exhibit a metabolic profile characterised by an intense energy crisis, severe oxidative damage, strong inflammatory responses, and significant metabolic abnormalities associated with impaired organ function (e.g., lungs and brain). These changes result from pathophysiological processes and may also contribute to disease progression. Therefore, investigating the relationship between the pathological phenotype of high-altitude illness and specific metabolic pathways and key metabolites is expected to reveal molecular targets for the early detection and treatment of high-altitude illness.

### 5.3 Mechanisms of metabolic regulation in high-altitude illness

**FIGURE 2 F2:**
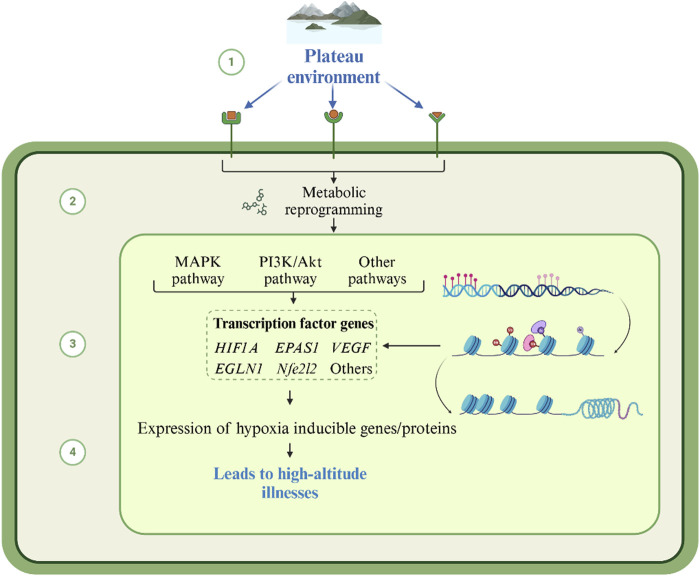
Mechanism of high-altitude illnesses caused by metabolite changes in the plateau environment. Under plateau hypoxia, accumulated metabolites regulate the function of target proteins through direct modification or alteration. This further triggers an imbalance in metabolic pathway networks and redistribution of metabolic flows, extending to the gut microbiota-host metabolic axis. This forms a multilevel positive feedback network.

Metabolites are not only biomarkers of environmental stress, but also “molecular switches” that actively drive plateau pathology by modifying biomolecules, reprogramming metabolic pathways, and reconfiguring the colony-host interaction axis (Figure 2). Although the association between high-altitude illness and metabolites has been increasingly studied, the mechanism of its dynamic regulatory network remains to be elucidated. Based on the available evidence, we propose a central hypothesis for the regulation of the metabolic cascade in high-altitude illness.

Metabolites drive the pathological processes of high-altitude illness through cascading regulatory networks in the low oxygen environment of the plateau. At the level of metabolite-biomolecule interactions, compensatory enhancement of anaerobic glycolysis leads to the accumulation of metabolites such as lactate, succinate semialdehyde, and hypoxanthine, which in turn modulate target protein function through direct modification or alteration. Lactate induces histone lactonylation, which opens chromatin regions of pro-inflammatory genes and initiates an early inflammatory storm. Accumulation of succinate semialdehyde competitively inhibits the corresponding transaminases and disrupts inhibitory neurotransmitter homeostasis, constituting a core toxicity mechanism in HACE. Hypoxanthine accumulation stimulates xanthine oxidase activity, catalyzes the explosive generation of superoxide anion, and lays the foundation for oxidative damage.

Perturbations at the metabolite-biomolecule level further trigger imbalances in metabolic pathway networks. Enhanced fatty acid oxidative compensation inhibits glycolytic pathways, reduces lysophosphatidylcholine synthesis, weakens cell membrane repair, and exacerbates tissue fragility. Purine metabolism collapses in response to accelerated erythropoiesis, ATP degradation drives uric acid accumulation, and superimposed lactate competitively inhibits renal excretion, which together activate inflammatory vesicles and drive systemic inflammation. Conversely, lipid-mediated storms manifest as hypermetabolism of arachidonic acid, which increases vascular permeability via receptors and directly drives the development of HAPE.

The redistribution of such metabolic flows extends to the gut microbiota-host metabolic axis. The plateau environment reshapes the metabolic profile of the bacterial flora, leading to a decrease in the abundance of microbiota, attenuation of protective butyrate synthesis, weakening of the intestinal barrier’s inhibitory effect on the HIF-1α pathway, and acceleration of pathologic erythropoiesis, which is the core pathological basis of CMS. Meanwhile, the inhibition of bacterial bile salt hydrolase caused bile acid dissociation disorder, which synergistically inhibited the respiratory chain of complex II with the mitochondrial toxicant fumaric acid, inducing energy failure and chronic fatigue.

Ultimately, the three levels of regulation intertwine to form a multilevel positive feedback network, with metabolite-modified pro-inflammatory proteins accelerating the release of lipid mediators, oxidative damage products inducing mitochondrial gene mutations, and sphingolipid remodeling pathways and inflammatory factors synergistically amplifying the damage signals to jointly promote the pathological triangle of the energy crisis-oxidative stress-inflammation cascade, which contributes to the irreversible transformation of plateau disease from acute reversible injury to chronic multi-organ failure. Therefore, the study of the association between the pathological phenotype of plateau diseases and specific metabolic pathways and key metabolites is expected to provide molecular targets for early warning and intervention of high-altitude illness.

## 6 Summary and outlook

Metabolism, a general term for chemical reactions in living organisms, is a fundamental characteristic of life. Metabolic regulation, in contrast, is a central event in cells and organisms that determines a series of critical processes, including cell growth, differentiation, functional maintenance, immune response, neuronal activation, memory storage, and tissue and organ development. Metabolic homeostasis regulation governs the selective activation and inhibition of specific metabolic pathways across cells, tissues, and organs at various developmental stages. It further determines their response to external factors, such as nutrition and hormones, under specific physiological and pathological conditions. Identifying the causes of metabolic disorders and their pathogenic mechanisms is currently a hot research topic and is relevant to the prevention and treatment of major diseases worldwide.

Diabetes mellitus, cancer, cardiovascular diseases, and cerebrovascular diseases are closely linked to metabolite dysregulation. In the low-oxygen conditions of the plateau, the potential relationship between susceptibility to high-altitude illnesses, plateau-related diseases, and associated metabolite imbalances warrants further investigation. Although evidence indicates that metabolite changes and altitude illnesses are related to the plateau environment, this relationship requires additional study. A deeper understanding of the specific mechanisms by which different metabolites regulate plateau diseases in the plateau environment, including their specific target genes, modes of action, and regulatory networks, is also needed.

With the advancement of technology and related research, we look forward to a deeper understanding of the interactions between metabolites and plateau diseases, which will provide more useful guidance for improving human health at high altitudes.
